# Effect of Spironolactone on COVID-19 in Patients With Underlying Liver Cirrhosis: A Nationwide Case-Control Study in South Korea

**DOI:** 10.3389/fmed.2021.629176

**Published:** 2021-02-23

**Authors:** Dongsub Jeon, Minkook Son, Jonggi Choi

**Affiliations:** ^1^Department of Gastroenterology, Liver Center, Asan Medical Center, University of Ulsan College of Medicine, Seoul, South Korea; ^2^Department of Biomedical Science and Engineering, Gwangju Institute of Science and Technology, Gwangju, South Korea

**Keywords:** coronavirus disease 2019, spironolactone, liver cirrhosis, infectivity, susceptibility

## Abstract

**Purpose:** On the basis that spironolactone is involved in ACE2 expression and TMPRSS2 activity, previous studies have suggested that spironolactone may influence the infectivity of COVID-19. Research has suggested that cell entry of SARS-CoV-2, the virus that induces COVID-19, is associated with the ACE2 receptor and TMPRSS2. The purpose of this study was to investigate whether spironolactone has a protective effect against COVID-19 and the development of associated complications in patients with liver cirrhosis.

**Methods:** We conducted a nationwide case-control study on liver cirrhosis patients with or without COVID-19 from the population-based data acquired from the National Health Insurance Systems of Republic of Korea. After 1:5 case-control matching, multivariable adjusted conditional logistic regression analysis was performed.

**Results:** Among the patients with liver cirrhosis, the case group with COVID-19 was found to be significantly less exposed to spironolactone compared with the control group without COVID-19. The adjusted odds ratio (OR) and 95% confidence interval (CI) between the two groups was 0.20 (0.07–0.54). In addition, regardless of cumulative dose of spironolactone, exposure to spironolactone was associated with lower COVID-19 infection. In terms of the development of complications due to COVID-19, spironolactone did not show any significant association between the patients with and without complications (*P* = 0.43). The adjusted OR and 95% CI between the two groups was 1.714 (0.246–11.938).

**Conclusion:** We conclude that spironolactone may reduce susceptibility to COVID-19 but does not affect the development of its associated complications; however, further studies are needed to confirm the exact association between spironolactone and COVID-19 infection.

## Introduction

The severe acute respiratory syndrome coronavirus-2 (SARS-CoV-2) is a novel coronavirus that causes coronavirus disease 2019 (COVID-19). COVID-19 has rapidly spread globally, and the World Health Organization declared COVID-19 a pandemic on March 11, 2020. The mortality rate based on cumulative data is around 3.4% in China and 0.4% outside of China (1). Despite the relatively low mortality rate, COVID-19 can cause severe complications such as acute respiratory distress syndrome (ARDS), with elderly patients being of particularly high risk (2).

Spironolactone is used primarily to treat heart failure, edematous conditions such as ascites in severe liver diseases, secondary hyperaldosteronism due to liver cirrhosis, and essential hypertension (3). The pharmacodynamics of spironolactone are diverse; for example, it is a mineralocorticoid receptor antagonist that tends to disclose favorable patterns of renin-angiotensin-aldosterone system (RAAS) and angiotensin-converting enzyme-2 (ACE2) expression. It also reduces transmembrane serine protease 2 (TMPRSS2) activity through its antiandrogenic activity (4–6). Previous studies have noted that cell penetration of SARS-CoV-2 is associated with the ACE2 receptor and TMPRSS2 (7–9). Research has therefore suggested that spironolactone may influence the infectivity of COVID-19 (4, 10, 11).

In light of this theory, we have conducted a nationwide case-control study investigating whether spironolactone exposure could be associated with SARS-CoV-2's infectivity and complication rate in COVID-19 patients with liver cirrhosis. The null hypothesis was that there are no differences between patients with or without spironolactone exposure in terms of SARS-CoV-2's infectivity and complication rate of COVID-19.

## Materials and Methods

### Data Source and Study Population

**Figure 1:**
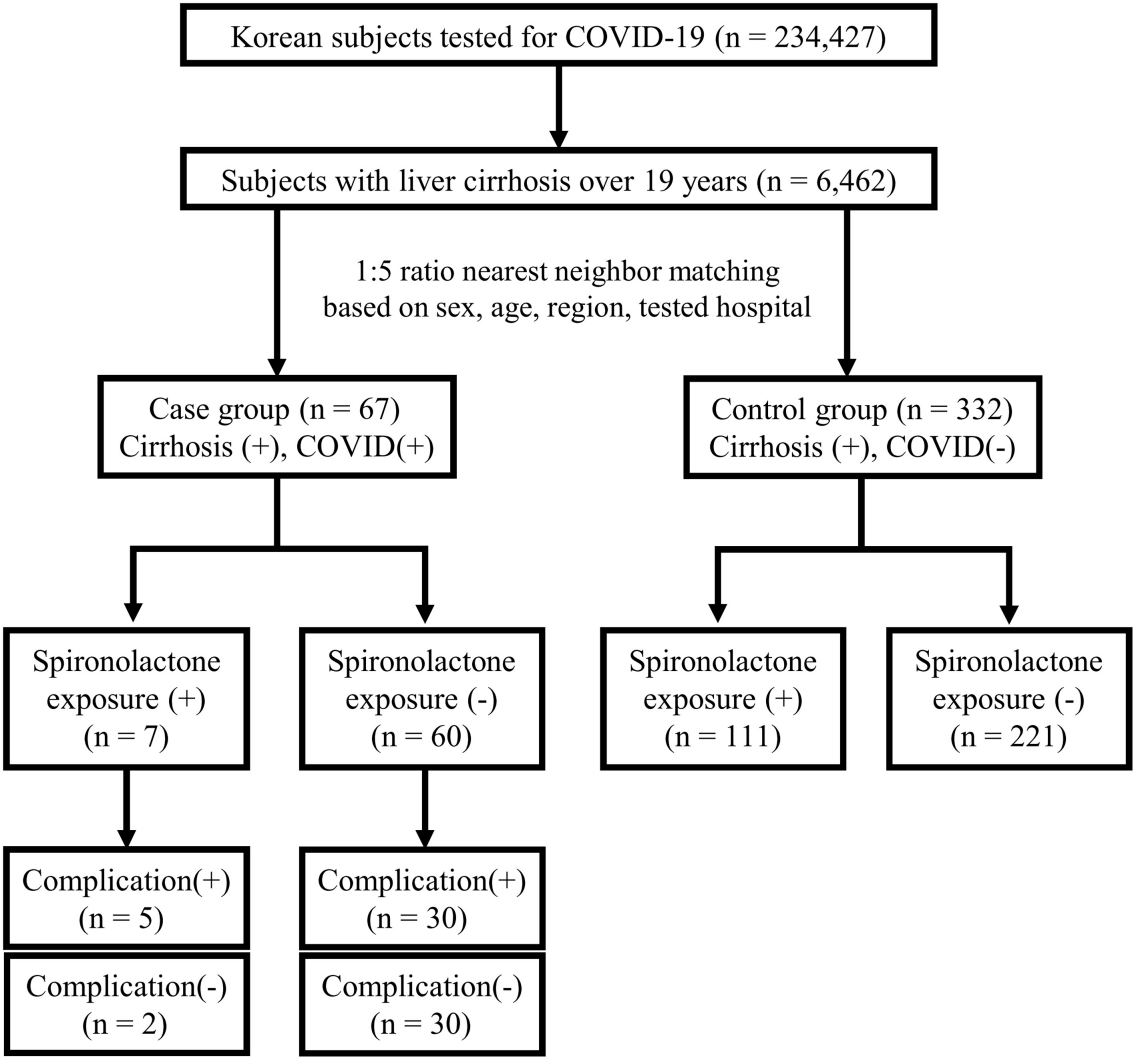
Study flow diagram.

This study was approved by the Institutional Review Board of Asan Medical Center (IRB number: 2020-1153) and written informed consent was waived by the board due to the de-identified nature of the data. The anonymized data obtained from the National Health Insurance claims of Republic of Korea were analyzed. The flow of the population in this case-control study is represented in Figure 1.

In detail, the population-based dataset comprised all patients tested for COVID-19 from January 20, 2020, when the first case of COVID-19 was observed in South Korea, to May 15, 2020, including suspected and confirmed cases, with demographic information and medical services history for the past 3 years. The analysis was performed on 234,427 patients tested for COVID-19 with the 10th revision of the International Statistical Classification of Diseases and Related Health Problems (ICD-10) diagnosis codes of B342, B972, Z208, Z290, U18, U181, Z038, Z115, U071, and U072. Screening was conducted by performing polymerase chain reaction amplification of the viral E gene and the RdRp region of the ORF1b gene was amplified to confirm COVID-19. Among the total 234,427 patients with COVID-19 screening test results, 6,462 subjects were confirmed to have liver cirrhosis over 19 years. The presence of liver cirrhosis was established based on ICD-10 codes for liver cirrhosis (K702, K703, K704, K717, K720, K721, K729, K740-K746, K761, K766-K767, R18, I850, I859, I864, I868, I982, I983) (12). Among patients with liver cirrhosis, there were 67 (1.0%) confirmed COVID-19 cases in the case group and 6,395 (99.0%) uninfected cases in the control group. Cases and controls were matched according to a 1:5 ratio based on covariates such as sex, age, region, and tested hospital, considering the explosive outbreak in Daegu and Gyeongbuk regions (13, 14). Patients were classified to either Daegu and Gyeongbuk regions or other regions, and hospitals in which patients had been tested were classified to tertiary hospitals and others. Patients' covariates were matched, but the nearest neighbor matching was performed on age, with a caliper width of 0.1 in propensity scores. The final numbers of cases and controls were 67 and 332, respectively. Then, whether the subjects were exposed to spironolactone within 1 year from when the patients were tested for COVID-19 was evaluated.

Further subgroup analysis for complication rate was done on the case group. Complications due to severe COVID-19 disease were defined as cases requiring intervention, such as oxygen therapy, anti-viral therapy, vasopressors, admission to the intensive care unit, continuous renal replacement therapy, or death (15) (Supplementary Table 1). Patients were divided into two groups: those with complications and those without complications (16). There were 35 and 32 patients with and without complications, respectively.

### Exposure to Spironolactone

Exposure to spironolactone was defined as the administration of spironolactone at least once within 1 year before the date of COVID-19 testing. Two additional sensitivity analyses were performed to verify the robustness of the study findings. With at least one claim within 6 months and 3 months for prescription of spironolactone, we classified these according to exposure to spironolactone and performed additional analyses. In addition, to quantify the exposure to spironolactone and to determine the dose-response association, the cumulative defined daily dose (cDDD) of spironolactone during the exposure period was calculated (≤30 cDDD or >30 cDDD) (17). The DDD was used for measuring a prescribed amount of a given drug and was considered the assumed average daily maintenance dose of a drug according to its main indication in adults (determined from the ATC/DDD system of the WHO Collaborating Center for Drug Statistics and Methodology) (18). For spironolactone, the WHO DDD is 75 mg. cDDD was calculated as the total amount of drug divided by the amount of that drug in DDD. The illustration for the study design and spironolactone exposure is presented in Supplementary Figure 1.

### Definitions of Covariates

Underlying diseases were established based on diagnosis codes of the ICD-10. The considered comorbidities were decompensated liver cirrhosis, diabetes, hypertension, dyslipidemia, cardiovascular disease including myocardial infarction and stroke, cancer, lung disease including chronic obstructive pulmonary disease and asthma, end-stage renal disease (ESRD) with dialysis, and immunocompromised status including autoimmune diseases and human immunodeficiency virus infections. These comorbidities in the present study were chosen based on the announcement of Centers for Disease Control and Prevention in the U.S that these comorbidities increased risk of severe illness from COVID-19 infection (19) (Supplementary Table 1) The Charlson Comorbidity Index (CCI) was also used as a covariate (20), and a higher CCI score indicated a greater likelihood that the predicted outcome would result in mortality.

### Statistical Analysis

Baseline characteristics of case and control groups were presented as mean with standard deviation for continuous variables, and the number with percentage (%) for categorical variables. Comparisons between both groups were performed using Student's *t*-tests for continuous variables and chi-squared or Fisher's exact tests for categorical variables. After 1:5 ratio case-control matching, the odds ratio (OR) and 95% confidence interval (CI) were calculated with conditional logistic regression analyses. For multivariable-adjusted analysis according to COVID-19 status, two models were used because of the limited study population. Model 1 was adjusted for hypertension, dyslipidemia, and CCI because CCI does not include hypertension and dyslipidemia. Model 2 was adjusted for decompensated liver cirrhosis, hypertension, cardiovascular disease, cancer, lung disease, ESRD with dialysis, and CCI, which were significant at the *P* < 0.10 level for the univariable analysis. Subgroup analysis was performed for COVID-19 status by dividing the study group by sex (male and female) and age (age ≥60 and <60 years). For multivariable-adjusted analysis according to the presence of complications, the model was adjusted for age, diabetes, hypertension, cancer, and CCI, which were significant at the *P* < 0.10 level in univariable analysis. The statistical software SAS for version 9.4 (SAS Inc., Cary, NC, USA) was used to perform all statistical analyses. A *P* < 0.05 was considered to be statistically significant.

## Results

### Baseline Characteristics

Before matching, the number of patients in the case and control groups were 67 and 6,395, respectively. After matching, a total of 399 subjects were analyzed. The baseline characteristics of the study population are presented in Table 1. The mean age was 60.2 years, and the proportion of male subjects was 59.4%. The proportions of decompensated liver cirrhosis, hypertension, cardiovascular disease, cancer, lung disease, and ESRD with dialysis were significantly higher in the control group compared with the case group. The CCI was higher in the control group than case group (6.3 vs. 4.3). The complication rate was 52.2% in the case group and 16.6% in the control group (*P* < 0.0001). Among complications, the presence of oxygen therapy and anti-viral therapy was significantly higher in the case group. The proportion of spironolactone exposure was 10.5% in the case group and 33.4% in the control group (*P* = 0.0002). Of the patients exposed to spironolactone, four case and 60 control patients had a spironolactone cDDD of >30, whereas, three case and 51 control patients had a spironolactone cDDD of ≤30.

**Table 1:** Baseline characteristics of patients with liver cirrhosis, according to COVID-19.

**Total (*n* = 399)**	**Patients with liver cirrhosis and COVID-19 (*n* = 67)**	**Patients with liver cirrhosis but not COVID-19 (*n* = 332)**	***P*-value**
**Demographics**			
Sex, male, n (%)	40 (59.7)	197 (59.3)	1.00
Age (years), mean (SD)	59.9 (15.7)	60.3 (15.3)	0.85
**Region of diagnosis**			
Daegu and Gyeongbuk, n (%)	43 (64.2)	212 (63.9)	1.00
**Tested hospital**			
Tertiary hospital, n (%)	9 (13.4)	45 (13.6)	0.98
**Comorbidities**			
Decompensated liver cirrhosis, n (%)	19 (28.4)	154 (46.4)	0.01
Diabetes, n (%)	21 (31.3)	121 (36.5)	0.43
Hypertension, n (%)	27 (40.3)	185 (55.7)	0.02
Dyslipidemia, n (%)	19 (28.4)	127 (38.3)	0.13
Cardiovascular disease, n (%)	9 (13.4)	82 (24.7)	0.04
Cancer, n (%)	12 (17.9)	113 (34.0)	0.01
Lung disease, n (%)	17 (25.4)	120 (36.1)	0.09
ESRD with dialysis, n (%)	0 (0)	21 (6.3)	0.03
Immunocompromised status, n (%)	9 (13.4)	31 (9.3)	0.31
Charlson Comorbidity Index, mean (SD)	4.3 (2.7)	6.3 (3.8)	<0.0001
**Complications**	35 (52.2)	55 (16.6)	<0.0001
Oxygen therapy, n (%)	12 (17.9)	32 (9.6)	0.04
Antiviral therapy, n (%)	28 (41.8)	1 (0.3)	<0.0001
Vasopressors, n (%)	4 (6.0)	14 (4.2)	0.52
Admission to the intensive care unit, n (%)	2 (3.0)	9 (2.7)	1.00
Continuous renal replacement therapy, n (%)	1 (1.5)	1 (0.3)	0.31
Death, n (%)	6 (9.0)	32 (9.6)	0.86
**Exposure to spironolactone**	7 (10.5)	111 (33.4)	0.0002
Non-user	60 (89.5)	221 (66.5)	0.0008
cDDD ≤30	3 (4.5)	51 (15.4)	
cDDD >30	4 (6.0)	60 (18.1)	

*ESRD, end-stage renal disease; SD, standard deviation; cDDD, cumulative defined daily dose*.

### Association Between Exposure to Spironolactone and Risk of Infection With COVID-19

The results of the logistic regression analysis for COVID-19 infection according to exposure to spironolactone are shown in Table 2. The adjusted OR (95% CI) in model 2 for COVID-19 between patients who were and were not exposed to spironolactone within 1 year was 0.20 (0.07–0.54). Additional analyses within 6 months and 3 months also show a significant difference between case and control groups (*P* < 0.05). Using non-users as reference, the adjusted ORs for patients with a spironolactone cDDD of ≤30 and >30 were significant regardless of different definitions for the timing of spironolactone exposure. However, a dose-response relationship was not shown for the association between spironolactone exposure and COVID-19 (Table 2).

**Table 2:** Odds ratios and 95% confidence intervals for COVID-19 according to exposure to spironolactone.

	**Case (%)**	**Control (%)**	**Crude OR (95% CI)**	***P*-value**	Adjusted OR* (95% CI)	***P*-value**	**Adjusted OR^†^ (95% CI)**	***P*-value**
**Within 1 year**								
Total	67 (100)	332 (100)						
Without exposure to spironolactone	60 (89.5)	221 (66.6)	1.00		1.00		1.00	
Exposure to Spironolactone	7 (10.5)	111 (33.4)	0.19 (0.08–0.47)	0.0003	0.21 (0.08–0.55)	0.001	0.20 (0.07–0.54)	0.002
**cDDD for spironolactone**								
Non-user	60 (89.5)	221 (66.5)	1.00		1.00		1.00	
cDDD ≤30	3 (4.5)	51 (15.4)	0.22 (0.07–0.72)	0.01	0.25 (0.08–0.86)	0.03	0.23 (0.07–0.78)	0.02
cDDD >30	4 (6.0)	60 (18.1)	0.25 (0.09–0.70)	0.009	0.32 (0.11–0.93)	0.04	0.30 (0.10–0.89)	0.03
**Within 6 months**								
Total	58 (100)	287 (100)						
Without exposure to Spironolactone	52 (89.7)	187 (65.2)	1.00		1.00		1.00	
Exposure to Spironolactone	6 (10.3)	100 (34.8)	0.17 (0.06–0.45)	0.0004	0.198 (0.071–0.555)	0.002	0.17 (0.06–0.49)	0.001
**cDDD for spironolactone**								
Non-user	52 (89.6)	187 (65.1)	1.00		1.00		1.00	
cDDD ≤30	3 (5.2)	51 (17.8)	0.21 (0.06–0.71)	0.01	0.26 (0.07–0.88)	0.03	0.25 (0.07–0.87)	0.03
cDDD >30	3 (5.2)	49 (17.1)	0.22 (0.07–0.74)	0.01	0.27 (0.08–0.92)	0.04	0.27 (0.08–0.93)	0.04
**Within 3 months**								
Total	49 (100)	245 (100)						
Without exposure to Spironolactone	43 (87.8)	156 (63.7)	1.00		1.00		1.00	
Exposure to Spironolactone	6 (12.2)	89 (36.3)	0.22 (0.09–0.56)	0.002	0.26 (0.10–0.68)	0.006	0.23 (0.08–0.64)	0.005
**cDDD for spironolactone**								
Non-user	43 (87.8)	156 (63.7)	1.00		1.00		1.00	
cDDD ≤30	3 (6.1)	48 (19.6)	0.23 (0.07–0.76)	0.02	0.25 (0.07–0.86)	0.03	0.26 (0.08–0.90)	0.03
cDDD >30	3 (6.1)	41 (16.7)	0.27 (0.08–0.90)	0.03	0.31 (0.09–1.05)	0.06	0.28 (0.08–1.00)	0.05

* *Model 1: adjusted for hypertension, dyslipidemia, and Charlson Comorbidity Index*.

† *Model 2: adjusted for decompensated liver cirrhosis, hypertension, cardiovascular disease, cancer, lung disease, ESRD with dialysis, and Charlson comorbidity index*.

*OR, odds ratio; CI, confidence interval; ESRD, end-stage renal disease; cDDD, cumulative defined daily dose*.

### Subgroup Analysis for COVID-19 Status According to Sex and Age

For risk stratification, subgroup analyses for COVID-19 status were performed by stratifying the study population by sex and age. The results of these analyses are shown in Supplementary Table 2. Importantly, most of the adjusted ORs and 95% CIs were found to be significant, especially in men and patients over 60 years of age.

### Comparison Between the Complication and No Complication Groups of Patients With Liver Cirrhosis and COVID-19

Baseline characteristics of the complication and no complication groups of patients with liver cirrhosis and COVID-19 infection are shown in Table 3. The proportions of diabetes, hypertension, and cancer were significantly higher in the complication group than in the no complication group. There was no significant difference in the proportion of patients exposed to spironolactone between the complication and no complication groups (*P* = 0.43). The crude and adjusted ORs (95% CI) of spironolactone exposure for the development of COVID-19-related complications were 2.50 (0.45–13.91) and 1.714 (0.25–11.94), respectively.

**Table 3:** Baseline characteristics of patients with liver cirrhosis and COVID-19.

**Total (*n* = 67)**	**Patients with complications (*n* = 35)**	**Patients without complications (*n* = 32)**	***P*-value**
**Demographics**			
Sex, male, n (%)	21 (60.0)	19 (59.4)	0.96
Age (years), mean (SD)	63.5 (15.8)	56.0 (14.8)	0.05
**Region of diagnosis**			
Daegu and Gyeongbuk, n (%)	21 (60.0)	22 (68.8)	0.46
**Tested hospital**			
Tertiary hospital, n (%)	5 (14.3)	4 (12.5)	1.00
**Comorbidities**			
Decompensated liver cirrhosis, n (%)	13 (37.1)	6 (18.8)	0.10
Diabetes, n (%)	15 (42.9)	6 (18.8)	0.03
Hypertension, n (%)	19 (54.3)	8 (25.0)	0.01
Dyslipidemia, n (%)	9 (25.7)	10 (31.3)	0.62
Cardiovascular disease, n (%)	4 (11.4)	5 (15.6)	0.73
Cancer, n (%)	10 (28.6)	2 (6.3)	0.02
Lung disease, n (%)	10 (28.6)	7 (21.9)	0.53
ESRD with dialysis, n (%)	0 (0)	0 (0)	-
Immunocompromised status, n (%)	4 (11.4)	5 (15.6)	0.73
Charlson Comorbidity Index, mean (SD)	5.0 (2.9)	3.5 (2.3)	0.02
**Complications**			
Oxygen therapy, n (%)	12 (17.9)	–	–
Antiviral therapy, n (%)	28 (41.8)	–	–
Vasopressors, n (%)	4 (6.0)	–	–
Admission for intensive care unit, n (%)	2 (3.0)	–	–
Continuous renal replacement therapy, n (%)	1 (1.5)	–	–
Death, n (%)	6 (9.0)	–	–
**Exposure to spironolactone**	5 (14.3)	2 (6.3)	0.43
Non-user	30 (85.7)	30 (90.9)	0.12
cDDD ≤30	1 (2.9)	2 (9.1)	
cDDD >30	4 (11.4)	0 (0)	

*ESRD, end-stage renal disease; SD, standard deviation; cDDD, cumulative defined daily dose*.

## Discussion

To summarize, the results showed that a significantly low proportion of cirrhosis patients with COVID-19 had previous exposure to spironolactone. Spironolactone was not significantly associated with complications. The factors associated with complications in cirrhotic patients with COVID-19 were diabetes, hypertension, cancer, and CCI score. This result of high-risk factors coincides with those indicated in previous studies (21, 22). Therefore, the null hypothesis was partially accepted and partially rejected.

The value of our study is that it provides theoretical evidence for the role of spironolactone in terms of COVID-19 susceptibility. A previous study by Cadegiani et al. (4) has proposed that spironolactone may have protective effects against COVID-19. Cadegiani et al. suggested that spironolactone could be a plausible candidate for prophylactic and early treatment of COVID-19. This was based on the theory that spironolactone could avoid SARS-CoV-2 cell entry by modulation of ACE2 expression, decreasing viral priming by reducing TMPRSS2 activity, attenuating the damage caused by the overexpression of angiotensin II-AT-1 axis, and inducing anti-inflammatory effects in the lungs through pleiotropy. Our study has shown that patient cases with COVID-19 had statistically significant lower exposure to spironolactone compared with patients without COVID-19 in liver cirrhosis controls. Considering that decompensated liver cirrhosis, hypertension, cardiovascular disease, cancer, ESRD, and CCI were higher in patients without COVID-19, it can be concluded that spironolactone may have protective effects against SARS-CoV-2's infectivity.

In our study, the result showed that there were no statistically significant correlations between complication rate and spironolactone exposure. This result could be distorted because there were only 35 patients in the complication group, which were too small, and comorbidities were unequally distributed, specifically the significantly higher CCI score of the complication group compared with the no complication group, which could raise the complication rate. When baseline characteristics from previous studies were analyzed (diabetes, hypertension, cancer, and CCI) as risk factors for COVID-19 complications, they were higher in patients in the complication group compared with those in the without complication group (21, 22). For these reasons, the protective effect against COVID-19 complication of spironolactone could be masked.

We acknowledge the limitations of our study. First, we used data from national health insurance claims, which potentially caused some discrepancies between actual therapeutic practices. In addition, due to the nature of the present study, biases from the unequal distribution of comorbidities between the two groups might have affected the association between the use of spironolactone and COVID-19, despite statistical adjustments. Second, it was challenging to define ARDS, so complications induced by this condition included cases treated with oxygen therapy and other severe complications related to the disease. Third, the susceptibility of contagious diseases can be affected by multiple factors such as sociocultural factors, which can be difficult to anticipate. We were also not able to gather information regarding patients' lifestyle-related factors such as smoking and alcohol drinking, which might affect the outcome of our study. Additionally, there was a small number of COVID-19 cases in patients with liver cirrhosis. Moreover, our study lacked detailed information about severity or stage of liver cirrhosis. Therefore, our results should be interpreted with caution because only complications in patients with COVID-19 and liver cirrhosis, and whether these patients were exposed to spironolactone, were investigated. Our results should therefore be validated in a larger cohort study.

Our study is the first to investigate the impact of spironolactone on patient susceptibility to COVID-19, and the prevalence of its associated complications. Based on relevant statistical analysis, patients who were infected by COVID-19 with underlying liver cirrhosis showed significantly lower spironolactone exposure rate compared to patients who were not infected by COVID-19 with underlying liver cirrhosis. Therefore, our results suggested that exposure of spironolactone may reduce susceptibility to COVID-19 in patients with liver cirrhosis. Further studies are needed to confirm the exact association between spironolactone and COVID-19.

## Data Availability Statement

The datasets presented in this study can be found in online repositories. The names of the repository/repositories and accession number(s) can be found at: https://hira-covid19.net/.

## Ethics Statement

The studies involving human participants were reviewed and approved by Institutional Review Board of Asan Medical Center, Seoul, Republic of Korea (IRB number: 2020-1153). Written informed consent for participation was not required for this study in accordance with the national legislation and the institutional requirements.

## Author Contributions

DJ, MS, and JC were responsible for the conception and design of the study, acquisition, analysis and interpretation of the data, and drafting of the manuscript. MS performed the statistical analyses. All authors have full access to all data used in the study and take responsibility for the integrity of the data and the accuracy of the data analysis, and approved the final version of the manuscript.

## Conflict of Interest

The authors declare that the research was conducted in the absence of any commercial or financial relationships that could be construed as a potential conflict of interest.

## Abbreviations

ARDS: Acute respiratory distress syndrome
CCI: Charlson Comorbidity Index
CI: Confidence interval
ESRD: End-stage renal disease
OR: Odds ratio
RAAS: Renin-angiotensin-aldosterone system
DDD: Defined daily dose
SARS-CoV-2: Severe acute respiratory syndrome coronavirus-2.
