# Supplementary material for: Effect of Spironolactone on COVID-19 in Patients With Underlying Liver Cirrhosis: A Nationwide Case-Control Study in South Korea
Source: Front Med (Lausanne). 2021 Feb 23;8:629176. doi: 10.3389/fmed.2021.629176 (PMC7940540; doi:10.3389/fmed.2021.629176)
Supplement: Supplementary file 1 [file Table_1.DOCX]

**Supplementary Material**

- **Supplementary Figure 1.** Study design and exposure of spironolactone.
- **Supplementary Table 1.** Definition of the complications and comorbidities.
- **Supplementary Table 2.** Subgroup analysis for COVID-19 status according to sex and age.

**Supplement Figure 1. Study design and exposure of spironolactone.**


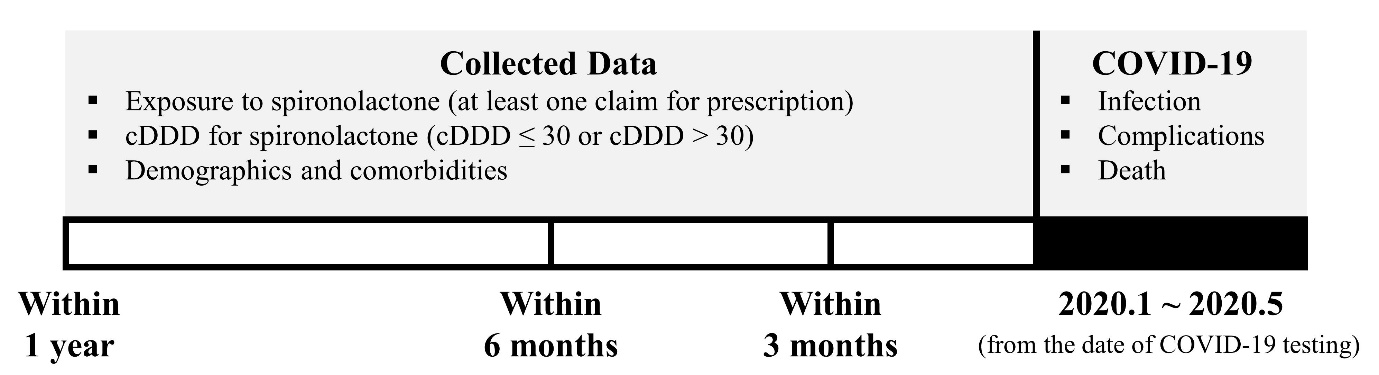


cDDD, cumulative defined daily dose.

**Supplementary Table 1. Definition of the complications and comorbidities.**

| **Complications** | **ICD-10 codes or HIRA general name code** | |
| --- | --- | --- |
| Oxygen therapy | M0040, M0046, M5850, M5857, M5858, M5859, M5860 | |
| Anti-viral therapy | Lopinavir/ritonavir: 4863  Darunavir: 4987, 6476  Interferon: 1755, 1757, 1758, 4526, 6544  Oseltamivir: 3589, 6469 | |
| Vasopressors | Norepinephrine: 2031  Epinephrine: 3138, 1526, 6694  Vasopressin: 2473  Dopamine: 1487, 3894, 3895, 3897, 4295  Dobutamine: 1482, 3890, 3996 | |
| Admission for intensive care unit | Claim codes including ‘AJ’ | |
| Continuous renal replacement therapy | O7031, O7032, O7033, O7034, O7035, O7051, O7052, O7053, O7054, O7055 | |
| **Comorbidities** | **ICD-10 codes** | **Claim codes** |
| Decompensated liver cirrhosis | K720, K721, K729, R18, I850, I983 | Admission or outpatient department ≥ 1 |
| Diabetes | E10-E14 | Prescription of anti-diabetic drugs ≥ 1 |
| Hypertension | I10-I11 | Prescription of anti-hypertensive drugs ≥ 1 |
| Dyslipidemia | E78 | Prescription of lipid-lowering drugs ≥ 1 |
| Cardiovascular disease | I21-I24 for myocardial infarction,  I60-I69 for stroke | Admission or outpatient department ≥ 1 |
| Cancer | C00-C97 | Admission or outpatient department ≥ 1 |
| Lung disease | J41-J44 for COPD,  J45-J46 for asthma | Admission or outpatient department ≥ 1 |
| ESRD with dialysis | N18-N19, Z49, Z99.2 | O701-O702 for hemodialysis ≥ 1 or  O707 for peritoneal dialysis ≥ 1 |
| Immunocompromised status | M05-M09, M30-M36 for autoimmune disease,  B20-B24, F24, O987, Z21 for HIV/AIDS | Admission or outpatient department ≥ 1 |

HIRA, Health Insurance Review and Assessment Service; COPD, chronic obstructive pulmonary disease; ESRD, end-stage renal disease.

**Supplementary Table 2. Subgroup analysis for COVID-19 status according to sex and age.**

|  | **Within 1 year** |  | **Within 6 months** |  | **Within 3 months** |  |
| --- | --- | --- | --- | --- | --- | --- |
| **Subgroup analysis for sex** | **Adjusted OR***  **(95% CI)** | **P-value** | **Adjusted OR***  **(95% CI)** | **P-value** | **Adjusted OR***  **(95% CI)** | **P-value** |
| **Total** | 0.20  (0.07–0.54) | 0.002 | 0.17  (0.06–0.49) | 0.001 | 0.23  (0.08–0.64) | 0.005 |
| Male | 0.34  (0.12–0.95) | 0.04 | 0.28  (0.09–0.90) | 0.03 | 0.24  (0.08–0.76) | 0.02 |
| Female | 0.19  (0.04–0.90) | 0.04 | 0.19  (0.04-0.89) | 0.03 | 0.34  (0.07–1.67) | 0.18 |
| **Subgroup analysis for age** | **Adjusted OR***  **(95% CI)** | **P-value** | **Adjusted OR***  **(95% CI)** | **P-value** | **Adjusted OR***  **(95% CI)** | **P-value** |
| **Total** | 0.20  (0.07–0.54) | 0.002 | 0.17  (0.06–0.49) | 0.001 | 0.23  (0.08–0.64) | 0.005 |
| Age ≥ 60 years | 0.33  (0.12–0.92) | 0.03 | 0.30  (0.10–0.96) | 0.04 | 0.31  (0.10–0.97) | 0.04 |
| Age < 60 years | 0.21  (0.05–0.93) | 0.04 | 0.23  (0.05–1.08) | 0.06 | 0.26  (0.05–1.25) | 0.09 |

*Adjusted for decompensated liver cirrhosis, hypertension, cardiovascular disease, cancer, lung disease, ESRD with dialysis, and Charlson comorbidity index.

OR, odds ratio; CI, confidence interval; ESRD, end-stage renal disease.
